# Cancer-associated fibroblasts and resistance to anticancer therapies: status, mechanisms, and countermeasures

**DOI:** 10.1186/s12935-022-02599-7

**Published:** 2022-04-29

**Authors:** Bing Feng, Jianzhong Wu, Bo Shen, Feng Jiang, Jifeng Feng

**Affiliations:** Jiangsu Cancer Hospital & Jiangsu Institute of Cancer Research & The Affiliated Cancer Hospital of Nanjing Medical University, 42 Baiziting, Nanjing, 210009 China

**Keywords:** Cancer-associated fibroblast (CAF), Tumor microenvironment (TME), Resistance, Chemotherapy, Immunotherapy

## Abstract

Cancer-associated fibroblasts (CAFs) are critical components of the tumor microenvironment (TME) with diverse functions such as extracellular matrix (ECM) remodeling, modulation of metabolism and angiogenesis, and crosstalk with both cancer cells and infiltrating immune cells by production of growth factors, cytokines, and chemokines. Within the TME milieu, CAFs exhibit morphological and functional transitions with relatively specific markers and hold tremendous potential to facilitate tumorigenesis, development, and resistance towards multiple therapeutic strategies including chemotherapy, radiotherapy, targeted therapy, anti-angiogenesis therapy, immunotherapy, and endocrine therapy. Accordingly, CAFs themselves and the downstream effectors and/or signaling pathways are potential targets for optimizing the sensitivity of anti-cancer therapies. This review aims to provide a detailed landscape of the role that CAFs play in conferring therapeutic resistance in different cancers and the underlying mechanisms. The translational and therapeutic perspectives of CAFs in the individualized treatment of malignant tumors are also discussed.

## Introduction

Cancer cells undergo uncontrolled proliferation and tendency of metastasis and therapeutic resistance owing to the support from complex tissue organizations in the tumor microenvironment (TME). The formation of these malignant phenotypes depends on both the genomic changes of cancer cells and the microenvironment suitable for their growth. The dynamic interactions between the tumor and TME profoundly influence the disease development and prognosis. In general, TME consists of cancer cells and heterogeneous nonmalignant constituents including fibroblasts, pericytes, immune cells, inflammatory cells, as well as soluble factors [1]. Activated fibroblasts, also known as cancer-associated fibroblasts (CAFs), are one of the well-recognized components of TME. Within the TME milieu, CAFs exhibit morphological and functional transitions and hold the tremendous potential to promote cancer progression as well as resistance to multiple therapeutics [2]. In this review, a thorough understanding of tumor and CAF crosstalk will be discussed. The functional role that CAFs play in conferring therapeutic resistance in different cancers and the underlying mechanisms have been exploited. The potential of novel markers for CAF-directed anticancer strategies will also be discussed.

**Fig. 1:**
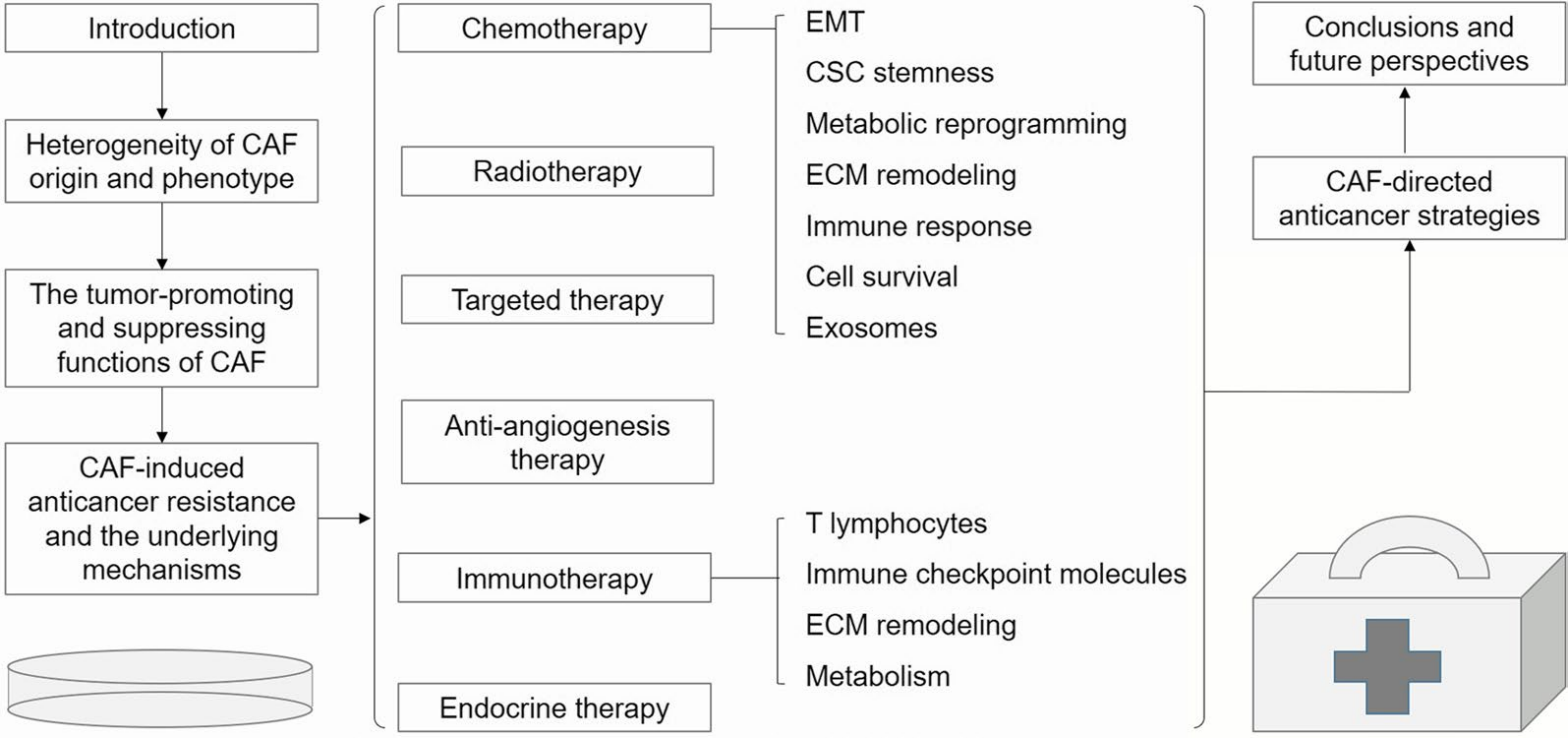
The outline of the manuscript. *CAF* cancer-associated fibroblast; *CSC* cancer stem cell; *ECM* extracellular matrix; *EMT* epithelial-mesenchymal transition

The outline of this review is shown in Fig. 1.

## Heterogeneity of CAF origin and phenotype

Emerging evidence indicates that CAFs originate from structural and functional alternations of heterogeneous cell populations upon the influence of various intrinsic and extrinsic factors. Tissue-resident fibroblasts, bone marrow-derived mesenchymal stem cells (MSCs), epithelial, and endothelial cells might transform into CAFs through transforming growth factor-β (TGF-β), epithelial-mesenchymal transition (EMT), or endothelial-mesenchymal transition (EndMT) [3]. CAFs can also evolve from the transdifferentiation of adipocytes or pericytes which leads to the upregulation of mesenchymal lineage-committed genes, such as *RUNX2* and *PPARγ* [4]. Moreover, vitamin deficiency in stromal cells might induce the upregulation of α-SMA and the differentiation into CAFs. In addition, CAFs can be derived from a variety of precursor cells recruited by cancer cells at both primary and metastatic sites including cancer stem cells (CSCs) [5].

Apart from the original heterogeneity, the diverse sources of CAF activation impact phenotypic heterogeneity. Histologically, the activated CAFs are spindled in shape with prominent nucleoli, rough endoplasmic reticulum, Golgi apparatus, gap junctions, and cytoplasmic myofilaments [6]. At this stage, the activated cells often show the expression of a broad range of distinct biological markers in a context-specific manner. Of note, although not exclusive to CAFs, the expression pattern of some surface markers such as alpha-smooth muscle actin (α-SMA), ferroptosis suppressor protein 1 (FSP1), secreted protein acidic and rich in cysteine (SPARC), platelet-derived growth factor β (PDGFβ), and integrin α 11, can be useful in the identification of CAFs [7].

## The tumor-promoting and suppressing functions of CAF

The primary role of CAFs is to remodel and regenerate the tissues in a highly-regulated, coordinating pattern. In the context of TME, CAFs facilitate tumorigenesis and cancer development by creating a pro-inflammatory, immuno-suppressive, and oxygen-rich microenvironment [8]. At the early stage of tumorigenesis, tumor-derived interleukin-1β induces CAF activation to orchestrate tumor-promoting inflammation in an NF-κB-dependent manner [9]. Furthermore, the immunosuppressive character of CAFs promotes tumor growth by facilitating immune evasion [10]. Some of the CAF subsets can deactivate the immune system directly by the expression of programmed death-ligand (PD-L)1/2 or the secretion of prostaglandin E2, an immunosuppressive factor that reduces the activation of T cells and NK cells [11]. Besides the direct effect on immune cells, CAFs take part in constructing extracellular matrix (ECM) protein networks that serve as a physical barrier for therapeutic drugs, as well as immune cells, from reaching the tumor [12]. Hypoxia is another well-known feature in the formation of the tumorigenic TME. In this context, CAFs often have upregulated expression of hypoxia-induced angiogenesis regulator (HIAR), which can increase CAF motility and secretion of vascular endothelial growth factor A (VEGFA) and further promote angiogenesis, thereby facilitating oxygenation and nutrient flow of the tumor [13].

In addition to their tumor-promoting role, CAFs are also involved in tumor suppression in some cases. For example, it is discovered that CAF ablation in genetically engineered mouse models of pancreatic ductal adenocarcinoma (PDAC) results in poorly differentiated tumors and shortened survival, indicating that sonic hedgehog (Shh)-driven CAFs can restrain tumor growth progression [14, 15]. Findings in bladder cancer and colon cancer also suggest that the Shh-Smo signaling-dependent CAFs are a source of urothelial differentiation factors [16, 17]. Further studies demonstrate that depletion of CAFs leads to invasive tumors and decreased survival *in vivo* with increased numbers of CSCs [18]. More recently, CD146 + CAFs, CAV1^high^ CAFs, and PDGFRα + Saa3- CAFs have been identified as tumor-suppressive CAF subsets in breast cancer [19]. Slit2 + and CD146 + CAFs suppress tumorigenesis and increase chemosensitivity, while molecules such as BMP4 reduce the self-renewal of stem-like cancer cells [20]. Meflin, a marker of MSCs, is recently identified as a functional contributor to cancer-restraining CAFs that counteract cancer-promoting CAFs in PDAC [21].

**Fig. 2:**
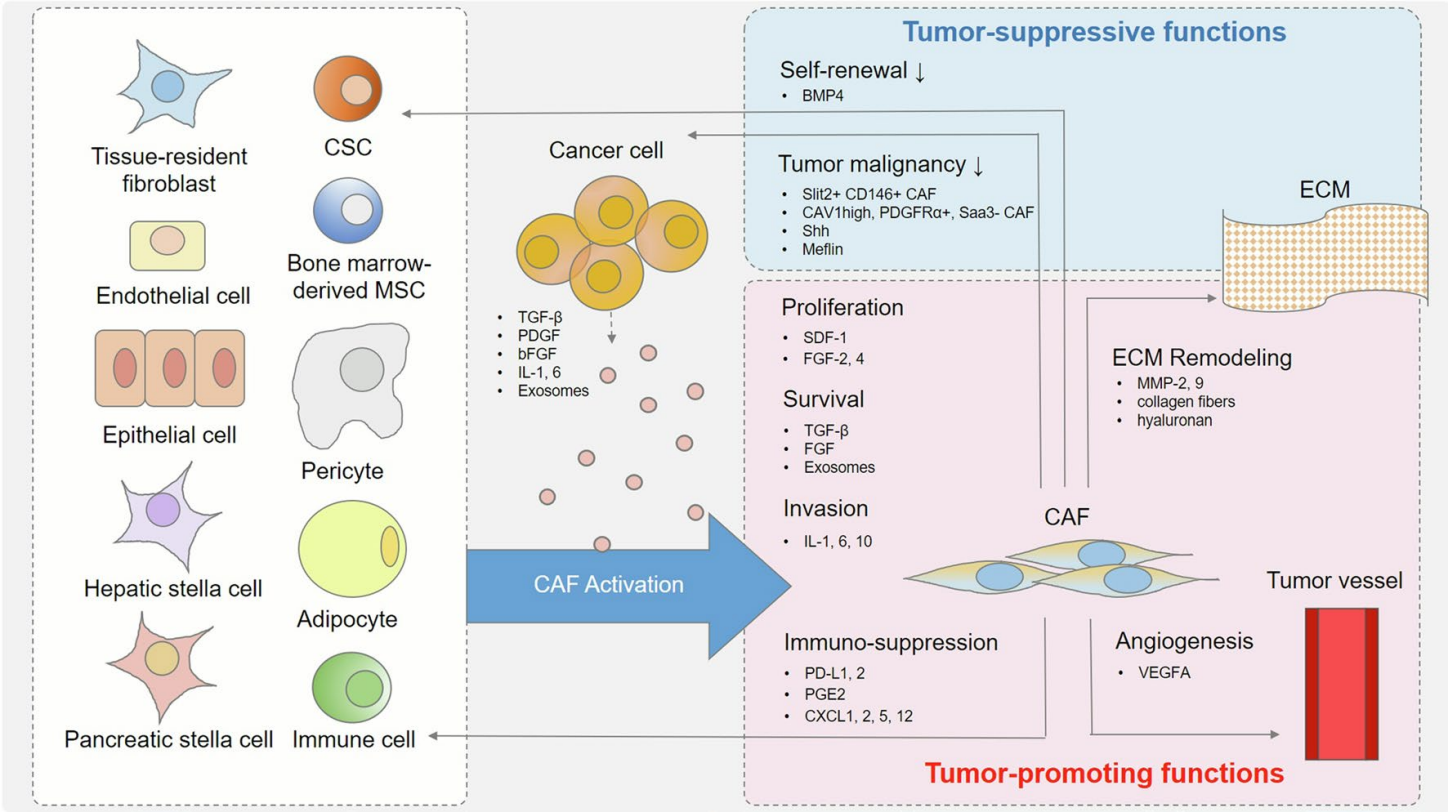
The original and functional heterogeneity of CAFs in tumor progression. CAFs originate from structural and functional alternations of heterogeneous cell populations upon the influence of various intrinsic and extrinsic factors. CAFs can either promote (red section) or suppress (blue section) cancer progression through multiple mechanisms by secreting numerous cell growth factors, cytokines, and chemokines. *BMP* bone morphogenetic protein; *CAF* cancer-associated fibroblast; *CSC* cancer stem cell; *CXCL* C-X-C motif chemokine ligand; *FGF* fibroblast growth factor; *IDO* indoleamine 2,3-dioxygenase; *IL* interleukin; *MMP* matrix metallopeptidase; *MSC* mesenchymal stem cell; *PDGF* platelet-derived growth factor; *PD-L1* programmed cell death ligand 1; *PGE2* prostaglandin E2; *TGF* transforming growth factor; *VEGF* vascular endothelial growth factor

The original and functional heterogeneity of CAFs in tumor progression is shown in Fig. 2.

## CAF-induced anticancer resistance and the underlying mechanisms

As mentioned above, CAFs are highly interrelated with sensitivity to anticancer therapies. According to the taxonomy raised by Meads et al., CAF-mediated drug resistance can be broadly divided into soluble and secretory factor-mediated drug resistance (SFM-DR) and cell adhesion-mediated drug resistance (CAM-DR) [22]. The SFM-DR is mediated by CAF-produced cytokines, chemokines, growth factors, exosomes, and desmoplastic reactions, which protect cancer cells against drug-induced apoptosis, while the CAM-DR is mediated by the adhesion of cancer cell integrins to stromal fibroblasts or to components of the ECM, such as fibronectin, collagen, and laminin. On the one hand, with the help of the paracrine regulatory factors including cytokines (TGF-β, TNF-α, IL-1, etc.), chemokines, and growth factors secreted by CAFs and cancer cells, CAFs facilitate in architecting a milieu feasible for tumor angiogenesis, metastasis, and therapeutic resistance, hence favoring tumor growth [23]. On the other hand, as the main component of the TME, CAFs act as a therapeutic barrier to prevent anticancer drugs as well as immune cell infiltration in solid cancer treatment [24].

**Fig. 3:**
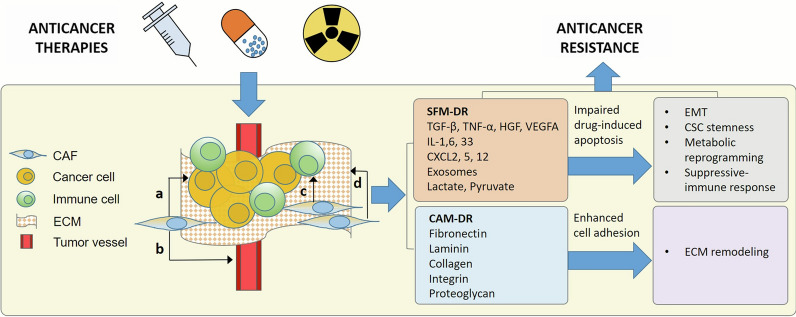
CAF-induced anticancer resistance. CAFs interact with cancer cells, immune cells, ECM, and tumor vessels in the TME, mediating drug resistance through either secretory factors or direct cell adhesion. **a** alternative pathways activating; **b** revascularization; **c** immunosuppression; **d** ECM remodeling. *CAF* cancer-associated fibroblast; *CAM-DR* cell adhesion-mediated drug resistance; *CSC* cancer stem cell; *CXCL* C–X–C motif chemokine ligand; *EMT* epithelial-mesenchymal transition; *HGF* hepatocyte growth factor; *SFM-DR* soluble and secretory factor-mediated drug resistance; *TGF* transforming growth factor; *TNF* tumor necrosis factor; *VEGF* vascular endothelial growth factor

The mechanisms by which CAFs are involved in anticancer resistance are shown in Fig. 3.

TGF-β is an extensively-studied, ubiquitously-expressed cytokine that plays a crucial role in tumor-stroma crosstalk. During the process of cancer progression, the dichotomous effect of CAFs is mainly manifested as an inhibitory factor in the early stage and a promoter in the advanced stage via the induction of diverse changes in CAFs, as well as the consequent secretion of diverse growth factors and cytokine including TGF-β [25]. CAF-induced drug resistance aided by TGF-β signaling will be described further in the corresponding sections underneath.

### Chemotherapy

Chemoresistance, both acquired and primary, is proven to be associated with complex multifactorial processes such as increased drug efflux, reduced drug uptake, activation of pro-survival signaling and defective apoptosis, acquisition of EMT and CSC-like properties, epigenetic modulation, as well as the interaction with the TME, especially CAFs [26].

#### CAF and EMT

EMT is a reversible process regulated by several EMT-related transcription factors (EMT-TFs) including ZEB, Snail, Slug, and Twist. Existing data suggest that the expression of EMT-TFs by CAFs is required for the paracrine stimulus on the adjacent cancer cells and is one of the critical factors involved in the development of chemoresistance [27]. For instance, in colon cancer, the expression of EMT-TF Snail1 in tumor stroma is correlated with similar expression levels in cancer cells, thereby promoting chemoresistance in cancer cells through EMT [28]. In PDAC, the EMT driver protein ZEB1 expression in CAFs is closely associated with prognosis as the only independent factor of survival after resection [29]. In breast cancer, TGF-β-induced CAFs upregulate the expression of EMT-TF Twist1 in the adjacent ER-positive cancer cells, influencing the aggressiveness and outcome of ER-positive breast cancers [30]. In another instance, CAF-secreted TGF-β1 increases the aggressiveness of breast cancer cells by activating the TGF-β/Smad signaling pathway, accompanied by enhanced migratory potential, invasiveness, as well as increased expression of mesenchymal markers such as MMP 2 and 9, vimentin, and fibronectin [31]. In ovarian cancer, TGF-β-induced CAFs instigate *VCAN* gene expression and EMT process, facilitating cancer cell motility, invasion, and drug resistance [32]. TGF-β can also regulate the expression of matrix metalloproteinases (MMPs), which are the key factors involved in EMT-related chemoresistance [33]. After being proteolytically activated by MMPs, TGF-β activates CAFs and further promotes fibrosis perpetuation as well as MMP expression and secretion [34]. In bladder cancer, CAF-derived TGF-β1 induces the expression of EMT-specific markers, such as ZEB2 proteins, in cancer cells and increases cancer invasiveness through ZEB2NAT transcript [35].

#### CAF and CSC stemness

CSCs are cancer cells with the capabilities of self-renewal, differentiation, clonal-tumor induction, and tumor immortalization [36]. The most important functions of CSCs are their role in resistance to multiple anticancer therapies and the repopulation of cancer cells after the treatment. Accumulating evidence indicates that with the stimulation of chemotherapy drugs, a variety of cytokines and chemokines derived from CAFs will participate in CSC maintenance, further promoting drug resistance. It has been reported that human CAFs treated with chemotherapeutic drugs induce CSC self-renewal and tumor growth in vivo with the concurrent release of cytokine IL-17 A in colorectal cancer [37]. In response to the chemotherapeutic treatment, the upregulated TGF-β signaling in the CAFs supports the tumor-initiating potential of CSCs, while TGF-β suppression blocks tumor-stroma crosstalk and leads to the attenuation of cancer progression [38]. In gastric cancer, CAFs increase the self-renewal of CSCs by secretion of neuregulin1 (NRG1) and activation of the downstream NF-κB signaling pathway, giving rise to enhanced proliferation and drug resistance to doxorubicin [39, 40]. Interestingly, it is demonstrated that EMT induction is often accompanied by the generation and amplification of CSCs. In prostate cancer, the switch from non-CSCs to CSCs, as well as chemoresistance, can be triggered by a hypomethylating event at CpG islands that induces the activation of genes involved in the main pathways of cell stemness, such as Hedgehog, Wnt, and NOTCH. CAF-induced hypomethylation of CGIs is correlated with the induction of EMT and stemness [41].

#### CAF and metabolic reprogramming

Cancer metabolism is identified as one of the hallmarks of cancer [8]. Current findings indicate that CAFs facilitate cancer therapeutic resistance via the exchange of several metabolites and accelerations of specific programs on differentiation or metabolic switches [42]. For instance, the breast cancer cells could induce multidrug resistance mediator GPER translocation in CAFs, stimulating a novel estrogen/GPER/PKA/CREB signaling activation, thereby contributing to glycolytic CAFs for the production of energy-enriched pyruvate and lactate. The energy metabolic coupling between catabolic CAFs and anabolic cancer cells confers the breast cancer cells with multi-drug resistance by increasing mitochondrial activity [43]. Particularly, the lactate released by CAFs confers lower extracellular pH in the TME, which is associated with doxorubicin and paclitaxel resistance as well as the higher migratory potential of cancer cells [44]. In the case of prostate cancer, docetaxel-resistant cancer cells demonstrate a more efficient intake of glucose and lactate from the stromal cells than the sensitive cells and induce mitochondrial oxidative phosphorylation (OXPHOS) as a novel pattern of glycolysis [45]. Further studies demonstrate that upregulation of Ras signaling in CAFs is correlated with increased glutamine synthesis and subsequent macropinocytosis of extracellular fluid. The glutamine secreted from CAFs will then promote mitochondrial metabolism of cancer cells and lead to lethal tumor growth as well as therapeutic resistance against androgen signaling deprivation drugs [46]. It is recently reported that in non-small cell lung cancer (NSCLC), hypoxia-induced exosomes by the cisplatin-resistant cancer cells can deliver pyruvate kinase M2 (PKM2), a key mediator in the process of cancer cells converting glucose into lactic acid [47], to CAFs, leading to the release of pyruvate and lactate and subsequent chemoresistance [48]. Interestingly, it is demonstrated that oxidative stress promotes the TGF-β signaling in CAFs and increases the synthesis of glycolytic byproducts such as pyruvate, ketone bodies, and L-lactate. These metabolites fuel cancer cell growth and ultimately lead to increased tumorigenesis and therapeutic resistance [49].

#### CAF and ECM remodeling

Cancer ECM is generally denser and stiffer than the normal tissues [50], which could increase solid stress and interstitial fluid pressure in tumors to creating hypoxia and metabolic-stressed milieu with increased expression of anti-apoptotic proteins and drug-resistant signaling pathways, hence facilitating tumor growth, CSC phenotype, and therapeutic resistance [51]. Recent studies have revealed a strong correlation between CAF-derived ECM remodeling and cancer chemoresistance [52]. First of all, CAFs produce ECM proteins and generate desmoplasia and fibrosis in the tumor stroma to create a physical barrier between cancer cells and therapeutic drugs as well as immune cells. In addition, CAFs secrete metabolites to fuel cancer cell growth under hypoxic and undernourished conditions [53].

#### CAF and immune response

As the most important stromal component, CAFs are closely correlated with immune cells as a mechanism of therapeutic escape of cancer cells and the development of chemoresistance. It is demonstrated that the interaction between tumor-associated macrophages and CAFs could facilitate cancer cells to gain gemcitabine and paclitaxel resistance in pancreatic and breast cancer in an IGF-1/2-dependent manner [54, 55]. Natural killer (NK) cells are potent cancer cell killers, but exposure to TGF-β which is secreted by CAFs abrogates their cytotoxic activity via miR-183 mediated *DAP12* transcription interruption, hence promoting cell survival and chemoresistance [56]. By secreting soluble factors such as CXCLs, CAFs also take part in the recruitment of tumor-associated neutrophils which aid in shaping TME and enhancing cancer cell proliferation, migration, and chemoresistance [57]. Additionally, CAF could secrete diverse cytokines and induce cancer chemoresistance in a paracrine manner. For instance, IL-6 is one of the typical cytokines secreted by CAFs that render chemoresistance in NSCLC cells. In an in vitro model, IL-6 family cytokine oncostatin-M (OSM) induces cancer cell EMT and escape from the targeted drug-induced apoptosis in an OSM receptor (OSMR)/JAK1/STAT3-dependent manner [58]. The role of CAF-derived IL-6 in inducing chemoresistance is also confirmed associated with the JAK2/STAT3 signaling pathway in patient samples of ovarian cancer [59]. Similarly, in gastric cancer, CAF-derived IL-11 is capable of inducing chemoresistance and CSC maintenance via the JAK/STAT3/Bcl2 signaling pathway [60].

#### CAF and cell survival

Considering the mechanism of chemotherapeutic drugs, it is obvious that apoptosis blockade could facilitate cancer cell survival and therapeutic resistance. It is suggested by a recent study that tumor necrosis factor superfamily member 4 (TNFSF4) is significantly up-regulated in lung CAFs under stress environments including chemotherapy, irradiation, and hypoxia. TNFSF4 not only inhibits the apoptosis of lung adenocarcinoma cells but also promotes cisplatin resistance mainly through enhanced activity of the NF-κB/BCL-XL signaling pathway [61].

Autophagy is also reported to be involved in the induction of chemoresistance of cancer cells [62]. In breast cancer, autophagy induced high-mobility group box 1 (HMGB1) secretion from CAFs further mediates CAF-CSC interaction and promotes tumorigenesis and therapeutic resistance in a Toll-like receptor 4 (TLR4)-dependent pattern [63]. CAF-mediated cisplatin resistance is also reported in tongue cancer via autophagy activation in the CAFs [64]. In the hypoxic TME, reactive oxygen species (ROS)-induced autophagy displays a negative feedback regulation by eliminating the source of ROS and thus protecting CAFs from oxidative damage. Blockage of autophagy resensitizes these CAFs to cisplatin [65]. In colorectal cancer, CAFs positively influence the metabolism of cancer cells through their autophagy and oxidative stress pathway which are initially induced by the neighboring cancer cells [66]. In cholangiocarcinoma, both in vitro and in vivo experiments show that CAF-derived IL-6 impairs the autophagy-associated apoptotic response to 5-FU in cancer cells. Cholangiocarcinoma patients with low stromal IL-6 levels and active autophagy flux in the cancer cells have a better prognosis and more effective response to postoperative chemotherapy [67].

DNA damage response is a network of cellular pathways that sense, signal, and repair DNA damage [68]. ROS produced in tumor stroma under oxidative stress is commonly observed during carcinogenesis, triggering DNA damage and genomic instability of adjacent cells including CAFs [69]. In turn, the soluble factors secreted by these ROS-induced CAFs promote cell survival and therapeutic resistance of cancer cells in a paracrine manner. For example, serine protease inhibitor Kazal type I (SPINK1), a senescence-associated secretory phenotype (SASP) factor produced in human stromal cells after genotoxic treatment, primes the aggressiveness and chemoresistance of cancer cells [70]. In multiple myeloma, the DNA-damaging drug doxorubicin could trigger an ataxia-telangiectasia-mutated (ATM) kinase-dependent DDR in bone marrow stromal cells, leading to increased IL-6 secretion by CAFs and resistance of myeloma cells to doxorubicin-induced apoptosis [71]. In prostate cancer, DNA damage increases the expression of the Wnt family member WNT16B in CAFs mediated by NF-κB, which further attenuates the cytotoxic effects of chemotherapeutic drugs mitoxantrone and docetaxel, thereby promoting the aggressiveness of the cancer cells in vivo [72].

#### Exosomes in CAF-mediated drug resistance

As nano-sized membrane-bound vesicles, exosomes provide new means of intercellular communication by delivering various bioactive molecules, including proteins, lipids, and nucleic acids, as well as participating in tumor initiation and progression [73]. Cancer-derived exosomes can change the behavior of surrounding stromal cells and vice versa, ultimately creating a suitable microenvironment for tumor growth [74]. It is suggested that in colorectal cancer, CAF-derived exosomes prime the drug-resistant character of CSCs by mediating the activation of the Wnt signaling pathway [75]. Further study indicates that CAFs contribute to cancer stemness, EMT, metastasis, and 5-FU/L-OHP resistance by directly transferring exosomes to cancer cells, which leads to a significant increase of miR-92a-3p and activation of Wnt/β-catenin pathway, hence inhibiting mitochondrial apoptosis by directly inhibiting F-box and WD repeat domain-containing 7 (*FBXW7*) and modulator of apoptosis 1 (*MOAP1*) [76]. In gastric cancer, CAF-secreted exosomal miR-522 could facilitate acquired drug resistance by induction of ferroptosis via arachidonate lipoxygenase 15 (*ALOX15*) regulation [77]. In PDAC, CAFs exhibit intrinsic resistance to gemcitabine. CAF-exosomes contribute to gemcitabine resistance by transferring miR-106b to neighboring cancer cells and directly targeting tumor protein 53-induced nuclear protein 1 (*TP53INP1*) [78]. In the context of ovarian cancer, it is revealed that CAF-derived exosomes carrying overexpressed miR-98-5p could promote cisplatin resistance of cancer cells by downregulating cyclin-dependent kinase inhibitor 1 A (*CDKN1A*) [79].

### Radiotherapy

Both in vitro and in vivo studies have confirmed the inhibitory effect of CAFs on cancer radiation response by either direct or paracrine interaction. For instance, radiotherapy treatment upon CAFs leads to increased secretion of HGF and elevated phosphorylation of c-Met, the HGF receptor, facilitating the proliferation and metastasis of pancreatic cancer cells [80]. An elevation of CXCL12 secretion is also confirmed in irradiated CAFs, with a stimulating effect on pancreatic cancer cell migration, invasion, and EMT-related drug resistance [81]. As previously described [30–35, 38], both EMT and CSC stemness can be modulated by stromal TGF-β. It is demonstrated that TGF-β produced by radiation-treated CAFs not only promotes cancer cell migration and potential metastatic escape but also augments resistance to radiotherapy, hence contributing to the poor survival outcomes of patients [82, 83].

Similar to the development of chemoresistance, desmoplasia is also involved in radioresistance via integrin β1 and the downstream FAK and MAPK-AKT signaling pathways in cancer cells [84]. The hypoxic TME created by the desmoplastic reaction will further exacerbate the radioresistant feature of cancer cells [85]. In lung cancer, it is reported that CAFs produce IGF1/2, CXCL12, and β-hydroxybutyrate post-radiation, which are capable of increasing ROS expression and protein phosphatase 2 A (PP2A) activity, thereby inducing autophagy in cancer cells and promoting cell recovery from radiation-induced damage both in vitro and in vivo [86].

### Targeted therapy

An increasing number of evidence supports the idea that EMT is involved in the development of resistance against tyrosine kinase inhibitors (TKIs) such as erlotinib and gefitinib in NSCLC [87, 88]. TKI-resistant cancer cells have mesenchymal cell characteristics based on cell morphology and upregulation of EMT-related proteins such as Vimentin and N-cadherin. In the nucleus, upregulation of p120-catenin and its binding to the Kaiso factor initiate transcription by activating EMT transcription factors including ZEB1, Snail, Slug, and Twist. The silencing of p120-catenin not only reverses the EMT process but also resensitizes cancer cells to erlotinib [89].

CAFs also play an active metabolic role in adaptive resistance to TKIs. It is proposed that under prolonged treatment with TKIs, EGFR- or MET-addicted NSCLC cells display an increase in producing glycolysis and lactate. Secreted lactate is the key molecule instructing CAFs to produce HGF in a nuclear factor kB-dependent manner, activating MET-dependent signaling in cancer cells, and finally sustaining resistance to TKIs [90]. Another study demonstrates that CAFs significantly increase the expression and phosphorylation of Annexin A2 (ANXA2) by secretion of HGF and IGF-1 as well as activation of the corresponding receptors c-met and IGF-1R, hence regulating EMT and gefitinib resistance in a paracrine manner [87].

### Anti-angiogenesis therapy

CAFs take part in the resistance of anti-angiogenesis including sorafenib, sunitinib, and bevacizumab mainly through secreting different angiogenic factors such as VEGF in the hypoxic TME [91]. For instance, CAFs from anti-VEGF-resistant murine lymphoma could down-regulate drug response of the sensitive cancer cells through revascularization in a PDGF-C-dependent manner both in vitro and in vivo [92]. In PDAC, CAFs stimulate the invasion activity of cancer cells via paracrine IGF1/IGF1R signaling, especially under hypoxia [93]. A recent study utilizing mass spectrometry-based proteomic analysis of CAFs indicates that hypoxic human mammary CAFs promote angiogenesis in CAF endothelial cell co-cultures in vitro by altering their secretion of various pro-and anti-angiogenic factors. Being the most increased protein in an abundance of hypoxic CAFs, HIAR exercises its pro-angiogenic and pro-migratory functions by inducing secretion of VEGFA and consequently enhancing VEGF/VEGFR downstream signaling in the endothelial cells [13].

### Immunotherapy

In recent years, the successful application of immune checkpoint inhibitors (ICIs) of cytotoxic T-lymphocyte antigen-4 (CTLA-4), programmed cell death-1 (PD-1), and programmed cell death ligand 1 (PD-L1) in various advanced cancers has attracted widespread attention in the field of immuno-oncology [94]. In this process, CAFs exert their immunomodulatory functions via modulation of both the cancer cells and the infiltrated immune cells, as well as the crosstalk among the complex components of the ECM [95, 96].

#### CAF and T lymphocytes

CAF abundancy is commonly correlated with aggressive clinical phenotype and poor responses to anticancer immunotherapy. The direct effect of CAFs on immunomodulatory is the attenuation of the CD8 + T lymphocyte function as well as the increase of the content in FOXP3 + regulatory T cells (Tregs), which are critical in maintaining immune tolerance and homeostasis of the immune system [97]. CAF subset heterogeneity analysis further refines the correlation between CAFs and immune infiltration. For example, fibroblast activation protein-α (FAP-α) + CAFs can increase the survival of CD4 + CD25 + T lymphocytes by secreting CXCL-12 and further induce these T cells differentiation into CD4 + CD25 + FOXP3 + Tregs and increase their ability to inhibit CD4 + effector T cell proliferation, thereby contributing to a tumor-promoting microenvironment in breast cancer and ovarian cancer [98]. FAP + CAFs also express high levels of TGF-β [99], which reduces T lymphocyte cytotoxicity by specifically inhibiting the expression of cytolytic gene products including perforin, granzyme A and B, Fas ligand, and interferon γ (IFN γ) [100]. TGFβ1 also reduces the responsiveness of memory T cells by blocking CD28-TCR signaling [101]. Interestingly, TGFβ secreted by CAFs can induce T cell apoptosis and enhance CTLA-4 + Tregs polarization [102]. Another subtype named PDPN + CAFs can suppress the proliferation of effector T lymphocytes in a nitric oxide-dependent manner, while PDPN- FAP + CAFs are not immunosuppressive [103]. Moreover, a recent study suggests that CD8 + T cells fail to infiltrate CAF-rich tumors, instead of accumulating at the tumor margin, and upregulating the expression of CTLA-4, leading to the resistance to multiple immunotherapies such as therapeutic vaccination and αPD1 [104]. In addition, CAF-derived IL-33 facilitates breast cancer metastasis *in vivo* by instigating type-2 inflammation in the metastatic microenvironment and mediates the recruitment of eosinophils, neutrophils, and inflammatory monocytes to the metastases [105]. Another study reveals that in melanoma and breast and colon cancers, TLR and Nod2 signaling could increase MCP-1 and RANTES expression in both cancer cells and CAFs. These secreted proteins promote the recruitment, generation, and expansion of Th17 cells [106], an independent lineage of Th cells that further promote tumor growth through the IL-17/IL-6/STAT3 functional axis [107].

#### CAF and immune checkpoint molecules

Given that CAFs could strongly inhibit T-cell proliferation in a contact-independent manner, it is further demonstrated that in pancreatic cancer, CAFs express higher levels of the PD-1 ligands PD-L1 and PD-L2 in comparison with normal tissues [108]. Immunotherapy based on PD-L1 blockade could not prevent the interaction of PD-L2 and PD-1, thus high expression of PD-L2 in CAFs could be a new mechanism of immunoresistance. Interestingly, the non-metastatic NSCLC patients with PD-L1 + CAFs exhibit significantly prolonged relapse-free survival than those with PD-L1- CAFs, and the expression of PD-L1 in CAFs is reversibly regulated by environmental stimuli including IFN γ from activated lymphocytes [109]. Mechanically, CAFs induce the expression of immune checkpoints such as T cell immunoglobulin mucin-domain-containing-3 (Tim-3), PD-1, CTLA-4, and lymphocyte activation gene (LAG)-3 on CD4 + and CD8 + T-cells, leading to fewer IFN γ, TNF-α, and CD107a production and a diminished immune function [108]. Subsequent studies indicate that CAFs promote PD-L1 expression in cancer cells through CXCL5 or CXCL2 secretion [110, 111]. Besides, CAF-derived exosomal miR-92 could downregulate large tumor suppressor kinase 2 (*LATS2*), an important component of the Hippo signaling pathway, leading to increased Yes-associated protein 1 (YAP1) nuclear translocation, thereby enhancing PD-L1 transcription and impairing T cell proliferation in breast cancer [112]. Moreover, CAFs themselves can function as antigen-presenting cells and induce CD8 + T cell death in an antigen-specific manner via PD-L2 and FASL [113].

#### CAF and ECM remodeling

As mentioned above, CAFs primarily prevent the infiltration and migration of immune cells by remodeling the ECM to serve as a contact barrier between the immune cells and cancer cells [114]. The dense ECM could also prevent T cells from therapeutic PD-1 inhibitors, thereby promoting the resistance of cancer cells to immune checkpoint inhibitors. Integrin α11 is a stromal collagen receptor that could promote tumor growth and metastasis and is associated with the regulation of collagen stiffness in the ECM. In a xenograft model of NSCLC, integrin α11 is reported to regulate the expression of CAF-derived lysyl oxidase like-1 (LOXL1), a matrix cross-linking enzyme, hence supporting tumor growth and immunoresistance through collagen matrix remodeling and collagen fiber alignment both in vitro and in vivo [115].

#### Metabolism in CAF-mediated immunosuppression

In addition to direct interaction with T cells and indirect influence via ECM remodeling, CAFs also take advantage of metabolic reprogramming to regulate T cell immunosuppression. On the one hand, glucose consumption by glycolytic CAF decreases environmental glucose levels of the TME, thereby impairing effector T cell activity without affecting cancer cell survival, as cancer cells could use lactate and pyruvate released by CAF [116]. On the other hand, the release of lactate by glycolytic CAFs acts on CD4 + T cells and shapes T cell polarization by decreasing Th1 and increasing Treg content [117]. Moreover, CAFs impair T cell function through increased activity of amino acid degrading enzymes involved in the regulation of immune tolerance of tumors [118]. For example, upon stimulation with IFN γ, CAFs express IDO protein and exhibit functional IDO activity, resulting in tryptophan depletion and kynurenine production as a novel T-cell inhibitory effector mechanism [119]. CAFs also inhibit anti-tumor effector T cell responses through arginase II (ARG2), which converts arginine to ornithine, leading to a lack of arginine, as well as reduced lymphocyte infiltration and attenuated function. The presence of ARG2-expressing CAFs is proposed to be an indicator of poor prognosis and hypoxia in cancer tissue [120]. In addition, it is detected in cervical cancer that CAFs express higher levels of CD39 and CD73 ectonucleotidases in cell membranes compared with normal tissues, and this feature is associated with the capability of decreasing the proliferation, activation and effector functions of cytotoxic T-cells through the generation of high amounts of adenosine from the hydrolysis of ATP, ADP and AMP nucleotides [121]. In melanoma, CAFs impair cytotoxic T lymphocyte (CTL) activity and reveal a pivotal role played by arginase in this phenomenon. CAF-derived soluble factors not only reduce CD69 on the surface of activated CTLs, but also increase l-arginase activity and CXCL12 release. The high amounts of CXCL12 by CAFs can act as a chemorepellent, explaining at least partially the exclusion of CD8 + T cells from solid tumors [122].

### Endocrine therapy

Endocrine therapy has become the cornerstone of hormone-sensitive tumors such as hormone-receptor-positive breast cancer and prostate cancer, while resistance is also widely observed. In breast cancer, tamoxifen induces the upregulation of TP53-induced glycolysis and apoptosis regulator (*TIGAR*), a p53 regulated gene that protects cancer cells against the onset of stress-induced mitochondrial dysfunction and aerobic glycolysis. In a CAF co-culturing model of breast cancer, it was demonstrated that mitochondrial activity in epithelial cancer cells drives tamoxifen resistance [123]. Further studies confirm that soluble stromal factors and extracellular matrix components are also involved in protection against tamoxifen-induced cell death. In detail, CAF-derived soluble factors protect the epithelial cancer cells from tamoxifen-induced cell death via EGFR and MMPs upstream of PI3K/AKT. Exogenous fibronectin confers endocrine resistance through interaction with integrin β1 and activation of PI3K/AKT and MAPK/ERK1/2 pathways. Treatment with both CAF co-culturing and fibronectin leads to the phosphorylation of the estrogen receptor at serine-118, suggesting stromal factors as modulators of ER activity [124]. In prostate cancer, the tumor stroma is enriched in CAFs that secrete androgen receptor (AR)-activating factors, which modulate AR signaling in cancer cells after androgen deprivation therapy. Loss of CAF-dependent AR activation may be responsible for castration-resistant prostate cancer progression [125]. The glutamine secreted from the CAFs also promotes resistance against androgen signaling deprivation therapy in the prostate cancer cells [49].

## CAF-directed anticancer strategies

The tumor-promoting functions that CAFs exert during cancer development make them promising targets in anticancer therapies. CAF-directed anticancer strategies can be generally divided into targeting the tumor-promoting function of CAFs, the downstream effectors, and the normalization of CAF-activated phenotype.

Anti-CAF therapies have been primarily focused on CAF depletion by targeting specific surface markers. For example, FAP causes rapid hypoxic necrosis of both cancer and stromal cells in dependence on IFN γ and TNFα to facilitate anti-tumor T cell infiltration and function, bringing benefits in transplantable models of NSCLC and PDAC [10, 126]. Targeting of FAP + CAFs by oral DNA vaccine increases the intratumoral infiltration of both CD8 + T cells and chemotherapeutic drugs in multi-drug-resistant breast and colon cancer [127, 128]. Further strategies such as FAP-CAR-T cell therapy and FAP-targeted oncolytic adenovirus promote a specific immune attack against FAP + CAFs, upregulate pro-inflammatory cytokines, and increase antigen presentation, T cell function, as well as trafficking, leading to enhanced anti-tumor efficacy [129–131]. FAP5-DM1, a monoclonal antibody targeting FAP + CAFs, induces long-lasting suppression of tumor growth and complete regression in xenograft models of a series of cancers with no obvious toxicity [132]. More recently, it is demonstrated in breast cancer and lung cancer that targeting the CD10 + GPR77 + CAFs correlated with chemoresistance and poor survival by using a neutralizing monoclonal antibody against GPR77 could induce tumor formation and improves chemotherapy efficacy in vivo [133]. Considering the complexity of cancer development, anticancer therapies are designed as combinatorial strategies that target crucial mediators in the TME and achieve promising and inspiring results. For example, Simlukafusp alfa (FAP-IL2v, RO6874281/RG7461) is an immunocytokine comprising an antibody against fibroblast activation protein α (FAP) and an IL-2 variant with a retained affinity for IL-2Rβγ > IL-2 Rβγ and abolished binding to IL-2 Rα. It is proven to be a potent immunocytokine that potentiates the efficacy of different T- and NK-cell-based cancer immunotherapies both in vitro and in vivo [134]. Another CAF-targeting strategy is to revert the activated state of the pro-tumorigenic CAFs into a relatively quiescent state or a tumor-suppressive phenotype. Treatment with vitamin D induces stromal reprogramming that normalizes the activated phenotype of CAFs and inhibits inflammation and fibrosis, improving the uptake of chemotherapeutic drugs and survival of pancreatic stellate cells [135].

Novel agents have been proposed to target downstream effectors and/or signaling pathways of CAFs including CAF-derived cytokines and chemokines. For instance, agents targeting IL-6, IL-6R, and JAK/STAT3 signaling pathways downstream of IL-6 have been approved by the US Food and Drug Administration (FDA) in myeloproliferative diseases and autoimmune disorders in order to suppress the FAP + CAF-induced proinflammatory cytokines and pro-angiogenic factors, which increase cancer cell proliferation and metastasis and negatively regulate T cell and NK cytotoxic activity [136]. In addition to IL-6, therapeutic agents targeting TGF-β signaling could interfere with the CAF activation or reduce the CAF numbers, leading to inhibited tumor growth and an anti-tumor effect. It is recently reported that small molecule kinase inhibitor LY2109761 blocks TGF-βRI and TGF-βRII receptors, suppresses the synthesis of CTGF, and reduces the stromal component of the tumors, leading to a significant reduction in the hepatocellular carcinoma growth, intravasation, and metastatic dissemination [137]. Galunisertib (LY2157299 monohydrate) is another TGF-βRI kinase inhibitor that specifically downregulates the phosphorylation of SMAD2, abrogating activation of the canonical pathway. It has been investigated in patients with glioblastoma, hepatocellular carcinoma, and pancreatic cancer [138]. Interestingly, co-administration of anti-TGF-β agents along with anti-PD-L1 immunotherapeutic drugs successfully suppresses TGF-β signaling of CAFs, facilitating T cell infiltration in TME and enhancing anti-tumor immunity [139]. Similarly, the AMD3100 compound, which targets the CXCL12-CXCR4 axis and reverses FAP + CAFs, impels immunosuppression and anti-PD-L1 immunotherapy in pancreatic cancer [140]. In addition, targeting CAF-induced fibrosis with the anti-fibrotic agent tranilast leads to enhanced tumor growth and invasiveness, as well as the immunosuppressive role of CAFs, via the decreased presence of Tregs and enhanced cytotoxic T cell response. This effect can be amplified in combination with effector-stimulatory immunotherapy such as dendritic cell-based vaccines [141].

Other anti-stromal therapies target ECM components in order to block cancer-driving signaling pathways and to facilitate the penetrance of therapeutic drugs as well as anti-tumor immune cells. MMPs and a disintegrin and metalloproteases (ADAMs) are the main metalloproteinase families participating in the remodeling of the ECM. Clinical trials have been undertaken with selective MMP and ADAM inhibitors, which are developed based on antibodies/antibody fragments or small molecules designed to take advantage of protease secondary binding sites or allosteric sites [142]. Moreover, it is demonstrated that the angiotensin inhibitor losartan could reduce stromal collagen and hyaluronan production and facilitate increased vascular perfusion, associated with decreased expression of profibrotic signals TGF-β1. Through this mechanism, losartan improves drug and oxygen delivery to TME in breast and pancreatic cancer models [143]. Additionally, remodeling the stroma of hyaluronan-rich tumors by depletion of hyaluronan, a PEGylated recombinant hyaluronidase, improves the antitumor activity of paclitaxel in the SKOV3/HAS3 tumor model [144].

Active clinical trials targeting CAFs in cancers are summarized in Table 1. Details for trials with NCT numbers can be accessed on https://clinicaltrials.gov (accessed on 1 December 2021).

**Table 1:** Active clinical trials targeting CAFs in cancers

NCT number	Cancer type	Drug	Compatibe drug		Target/mechanism	Clinical phase	Refs.
NCT02699606	NSCLC, urothelial cancer, gastric cancer, esophageal cancer or cholangiocarcinoma	Erdafitinib		/	A pan- FGFR tyrosine kinase inhibitor	Phase 2	[145]
NCT03762122	Squamous-cell NSCLC	Rogaratinib		/	A pan-FGFR inhibitor	Phase 2	[146]
NCT03386721	Head and neck, oesophageal or cervical cancer	RO6874281		Atezolizumab (MPDL3280A), or Gemcitabine and Vinorelbine	An immunocytokine consisting of IL-2v targeting FAP-α	Phase 2	[147]
NCT02627274	Breast cancer or head and neck cancer	RO6874281		Trastuzumab or Cetuximab	An immunocytokine consisting of IL-2v targeting FAP-α	Phase 1	[148]
NCT03875079	Melanoma	RO6874281		Pembrolizumab	An immunocytokine consisting of IL-2v targeting FAP-α	Phase 1	[149]
NCT03834220	Solid tumors	Debio 1347		/	A pan-FGFR inhibitor	Phase 2	[150]
NCT03822117	Solid tumors	Pemigatinib		/	Targeting activating FGFR mutations or translocations (FIGHT-207)	Phase 2	[151]
NCT02872714	Urothelial cancer	Pemigatinib		/	Tarageting FGF/FGFR alterations (FIGHT-201)	Phase 2	[152]
NCT02924376	Cholangiocarcinoma	Pemigatinib		/	Targeting FGFR2	Phase 2	[153]
NCT02399215	Carcinoid tumor, metastatic carcinoid tumor, or neuroendocrine neoplasm	Nintedanib		/	Blocking VEGFR from attaching to its target	Phase 2	[154]
NCT02834780	Hepatocellular carcinoma	H3B-6527		/	Targeting FGFR4 and FGF19	Phase 1	[155]
NCT03343301	Gastrointestinal cancer	FPA144		mFOLFOX6	Targeting FGFR2	Phase 1	[156]
NCT02432274	Solid malignant tumors or osteosarcoma	Lenvatinib		Ifosfamide, and Etoposide	An inhibitor of VEGFR2 tyrosine kinase with potential antineoplastic activity	Phase 1/2	[157]
NCT02508467	Hepatocellular Carcinoma	Fisogatinib (BLU-554)		/	Targeting FGF19	Phase 1	[158]

*FAP* fibroblast activation protein; *FGF* fibroblast growth factor; *FGFR* fibroblast growth factor receptor; *IL-2v* interleukin-2 variant; *NSCLC* non-small-cell lung cancer; *VEGFR* vascular endothelial growth factor receptor

## Conclusions and future perspectives

In recent years, increasing evidence has demonstrated the participation and importance of CAFs in tumorigenesis, development, immunosuppression, and drug resistance in a variety of cancers. As a key component of TME, CAFs exercise phenotypical and functional heterogeneity in a context-dependent manner in close relationship with TME as well as the host as a whole. Current research hotspots of CAFs in tumorigenesis and therapeutic resistance are mainly focused on subgroup analysis and functional research relying on CAF-specific markers and secretions such as IFN γ and TGF-β, which are expressed at different levels at different cancer stages in a context-specific manner. Existing results show that some targeting markers used in CAF-directed anticancer strategies are actually non-specific and also found on cancer cells as well. Moreover, hypoxia, acidic microenvironment, and tumor vascular abnormality remain the hurdles to be overcome concurrently in practice. To this end, more reasonable in vitro and in vivo research models are being called for. From the strategic point of view, the development of combinatorial strategies which not only target the interplay between CAFs and the TME but also promote conventional therapeutic effectiveness is still the mainstream direction in clinical trial design. Furthermore, attention should be paid to the dose of combinatorial agents and the potential occurrences, in order to truly realize the individualized anticancer therapies in the future.

## Data Availability

Not applicable.

## Abbreviations

BMP: Bone morphogenetic protein
CAF: Cancer-associated fibroblast
CAM-DR: Cell adhesion mediated drug resistance
CSC: Cancer stem cell
CTL: Cytotoxic T lymphocyte
ECM: Extracellular matrix
EMT: Epithelial-mesenchymal transition
FAP: Fibroblast activation protein
FGF: Fibroblast growth factor
ICI: Immune checkpoint inhibitors
IFN: Interferon
IL: Interleukin
MSC: Mesenchymal stem cell
NK: Natural killer
NSCLC: Non-small cell lung cancer
PD-1: Programmed cell death-1
PD-L1: Programmed cell death ligand 1
PDAC: Pancreatic ductal adenocarcinoma
ROS: Reactive oxygen species
SFM-DR: Secretory factor-mediated drug resistance
Shh: Sonic hedgehog
TGF: Transforming growth factor
TKI: Tyrosine kinase inhibitors
TLR: Toll-like receptor
TME: Tumor microenvironment
Treg: Regulatory T cell
VEGFA: Vascular endothelial growth factor A
